# Atrophy and Other Potential Factors Affecting Long Term Deep Brain Stimulation Response: A Case Series

**DOI:** 10.1371/journal.pone.0111561

**Published:** 2014-10-31

**Authors:** Daniel Martinez-Ramirez, Takashi Morishita, Pamela R. Zeilman, Zhongxing Peng-Chen, Kelly D. Foote, Michael S. Okun

**Affiliations:** 1 Department of Neurology, University of Florida College of Medicine, Center for Movement Disorders and Neurorestoration, Gainesville, Florida, United States of America; 2 Department of Neurosurgery, University of Florida College of Medicine, Center for Movement Disorders and Neurorestoration, Gainesville, Florida, United States of America; Georgia Institute of Technology, United States of America

## Abstract

**Objective:**

To describe three DBS cases which presented with new side effects or loss of benefit from stimulation after long-term follow-up and to discuss the potential contributing factors.

**Methods:**

A University of Florida (UF) database (INFORM) search was performed, identifying three patients, two Parkinson's disease (PD) and one Essential Tremor (ET), with an unexpected change in long-term programming thresholds as compared to initial evaluation. Clinical follow-up, programming, imaging studies, and lead measurements were reviewed. The UF Institutional Review Board (IRB) approved this study.

**Results:**

A substantial increase in the 3^rd^ ventricular width (120%), Evans index (6%), ventricular index (5%), and cella media index (17%) was uncovered. A change in thresholds across lead contacts with a decrease in current densities as well as a relative lateral change of lead location was also observed. Hardware-related complications, lead migration, and impedance variability were not identified.

**Conclusions:**

Potential factors contributing to long-term side effects should be examined during a DBS troubleshooting assessment. Clinicians should be aware that in DBS therapy there is delivery of electricity to a changing brain, and atrophy may possibly affect DBS programming settings as part of long-term follow-up.

## Introduction

Deep Brain Stimulation (DBS) has become an effective treatment in select Parkinson's disease (PD), essential tremor (ET), and dystonia cohorts [1], [2], with motor benefits reported at 10 years following implantation [3], [4]. Several groups have suggested recommendations on how to manage and troubleshoot factors that may be responsible for a worse DBS outcome [5], [6] or factors leading to dissatisfaction during long-term management [7]. These issues, which have been previously described, include surgery-related complications (intracerebral hemorrhage, deep cerebral venous hemorrhage/infection, seizure, sterile seroma, pulmonary embolism, pneumonia, perioperative confusion, suboptimal lead placement), hardware-related, (infection, skin erosion, electrode or wire fracture, lead migration, neurostimulator malfunction, neurostimulator migration, pain in region of neurostimulator) and stimulation-related issues. Other possible factors include disease progression, poor selection of DBS candidates, unrealistic patient expectations, improper programming and medication adjustment, tolerance to DBS stimulation, and neuropsychiatric complications [8].

Because of the rapidly growing number of patients with DBS, it is critically important to recognize which factors, and how each of these factors will affect long-term response. We present three patients with previously unrecognized long-term DBS related management issues. These cases led to the investigation of potential mechanism(s) and factors for the stimulation-induced adverse events and loss of benefit.

## Methods

The University of Florida (UF) Institutional Review Board (IRB) approved the use of our IRB approved UF INFORM database. Three patients were identified in our database, two PD and one ET, with an unexpected change in long-term thresholds compared to initial evaluation performed 1 month after DBS surgery. The term thresholds, as used in this paper, will define persistent stimulation-induced adverse effects for each electrode in the monopolar mode. Patient records/information was anonymized and de-identified prior to analysis. Analysis included age, gender, diagnosis, disease duration, target, follow-up duration, DBS settings (voltage, pulse width, frequency, impedance, and current density), and Unified Parkinson's Disease Rating Scale (UPDRS) motor or Tolosa-Fahn-Mardsen Tremor Rating Scale (TRS) [9] scores at baseline, immediate postoperative, and long-term follow-up. Current density, which is a measurement of electrical flow, was calculated at 6 months post DBS surgery (once settings were optimized) and at 1 month after the most recent thresholds testing (using the settings tolerated by patients). The formula used was: (V x PW/impedance)/0.06 cm^2^ [10]. Initial programming thresholds were compared to a follow-up and imaging study was also obtained as part of troubleshooting evaluation.

### Lead location and atrophy indexes

Lead localization was measured using CT and MRI fused images. A possible lead migration was assessed applying Cartesian coordinates between two time points, measuring the electrode' tip in the post-operative scan, and comparing it to the most recent lead location (at the time of the side effect) using surgical planning software. Coordinates were assessed relative to the midcommissural point. Standard error measurement of deviation was set at 1.4 mm, as previously reported [11]. In order to assess ventricle size with respect to brain tissue and cerebral atrophy, different indexes (Evans index, ventricular index, cella media index, and maximum width of third ventricle) [12], [13] were obtained from pre-surgical imaging and compared to the most recent brain scan. The Evans index is defined as the maximal frontal horn ventricular width divided by the transverse inner diameter of the skull. The ventricular index is defined as the minimum width of the lateral ventricles divided by the maximum width of the anterior horns of the lateral ventricles. The cella media index is the ratio of biparietal diameter of skull to the maximum external diameter of lateral ventricles at cella media. The maximum width of the third ventricle was measured drawing a line through the long axis of the third ventricle, parallel to the interhemispheric fissure where the third ventricle was most visible. The width (in millimeters) was measured by drawing a second line perpendicular to the first line at its midpoint.

## Case Reports

### Case 1

A 50 year-old male with PD status post right subthalamic nucleus (STN) DBS implantation 75 months prior, complained of a subacute onset of left facial pulling that fully resolved with DBS deactivation. Surgery was initially indicated for “on state” disabling dyskinesias and tremors unresponsive to levodopa. Baseline pre-operative UPDRS motor score off/on meds was 62/16 with post-operative scores off med/on stim of 37 and on med/on stim of 31 at 6 months. During initial thresholds testing, the voltages required to produce left face/arm paresthesias were 2.9 for contact 0, 3.2 for contact 1, 3.0 for contact 2, and 3.8 for contact 3. Currently, the voltages required to produce muscle pulling, paresthesias, and cloudiness of thinking, were 0.5 for contact 0, 1.2 or contact 1, 1.2 for contact 2, and 0.9 for contact 3. His brain scan revealed an increase in the maximum width of the third ventricle compared to the baseline scan performed six years prior. Ten months after rechecking thresholds, the patient was doing well with lower settings and has complained of only one potential episode of facial pulling which was deemed by the clinical team likely unrelated to stimulation.

### Case 2

**Figure 1:**
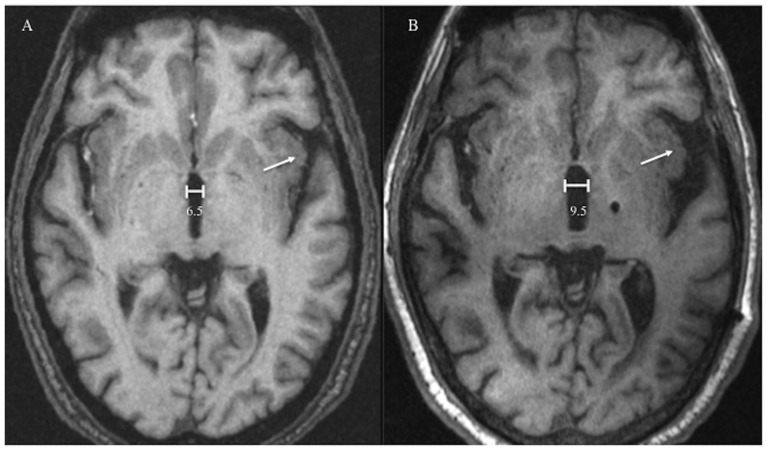
Comparison of two MRI scans over 10-year period. The post-operative scan 10 years after DBS surgery shows an increase in size of the ventricles (millimeters) and widening of the Sylvian fissure (arrow). A: Preoperative scan for DBS targeting; B: Most recent scan.

A 63 year-old male with PD status post left STN DBS implantation performed 99 months prior, presented with increased “off” time and re-emergence of troublesome dyskinesias over the last several weeks. The main indication for surgery was his “on state” dyskinesias and severe “off state” periods. His preoperative motor score off/on was 50/12 with post-operative scores off med/on stim of 35 and on med/on stim of 22 at 6 months. The levodopa equivalent daily dosage (LEDD) before surgery was of 1275 mg. During initial thresholds testing, the voltages required to produce right-sided paresthesias and right arm pulling were 2.0 for contact 0, 2.5 for contact 1, 4.0 for contact 2, and 6.0 for contact 3. Currently, the voltages required to produce right arm paresthesias were 0.5 for contact 0, 0.8 or contact 1, 1.5 for contact 2, and 0.8 for contact 3. LEDD at the time of the troubleshooting visit was of 762.5 mg. His MRI revealed an increase in size of the ventricles as compared to his baseline scan, performed 10 years before (Figure 1). Two years after re-programming with lower settings, the patient's symptoms have been controlled, but psychiatric issues have emerged.

### Case 3

A 65 year-old woman with ET status post left ventralis intermedius nucleus (Vim) nucleus DBS implantation 47 months prior presented with a subacute lack of benefit from DBS. Her indication for DBS included a long history of medically refractory tremor. The baseline TRS motor score was 46. During the initial thresholds testing, the voltages required to produce tingling of the upper extremity were 2.6 for contact 0, and 4.1 for contact 1; contacts 2 and 3 did not produce side effects at 6 or more volts. Currently, the voltages required to produce right arm paresthesias were 1.0 for contact 0, 1.0 or contact 1, 0.3 for contact 2, and 3.3 for contact 3. A recent scan revealed a marked increase in ventricular size as compared to the initial scan following DBS surgery. Twenty months after reprogramming, the patient reported feeling well, and no adjustment in settings have been required. Table 1 describes patient's characteristics with subsequent motor scores. Table 2 compares the initial with the most recent threshold testing, revealing decrements in the amount of voltage tolerated in cases, as well as decrements in current densities in two patients.

**Table 1:** Demographic and clinical characteristics of patients.

	Patient 1	Patient 2	Patient 3
**Gender**	Male	Male	Female
**Age, y**	50	63	65
**Diagnosis**	PD	PD	ET
**Disease duration, y**	17	24	>20
**Target**	R STN	L STN	L Vim
**Follow-up, mo.**	75	99	47
**UPDRS-III**			N/A
**Pre-op off/on**	62/16	50/12	
**Post-op off med/on stim**	27	35	
**Post-op on med/on stim**	21	22	
**5 y follow-up**			
**off med/on stim**	19	33	
**on med/on stim**	13	22	
**8 y follow-up**	N/A		
**off med/on stim**		41	
**on med/on stim**		21	
**TRS motor**	N/A	N/A	
**Pre-operative**			46
**4 y off/on stim**			26/25

PD = Parkinson's disease; ET = Essential Tremor; R = Right; L = Left; STN = subthalamic nucleus; Vim = ventralis intermedius nucleus; UPDRS = Unified Parkinson's Disease Rating Scale; TRS = Tremor Rating Scale.

**Table 2:** Comparison between initial and current thresholds.

	Contact	Initial (Voltage – Side effect)	Currently (Voltage – Side effect)	Δ Volts	Δ Time (months)
**Patient 1**	**0**	2.9 – tingling left hand	0.5 – bilateral hand tingling	−2.4	75
	**1**	3.2 – pulling face, cheek, jaw	1.2 – left neck and face pulling	−2.0	
	**2**	3.0 – tingling left face/arm	1.2 – cloudiness of thinking	−1.8	
	**3**	3.8 – tingling left face/arm	0.9 – pulling left face and tongue	−2.9	
	**Impedance**	1291 Ω	Postop - 1285 Ω; Chronic - 747 Ω		
	**Current density**	3.15 µC/cm^2^/phase	1.99 µC/cm^2^/phase		
**Patient 2**	**0**	2.0 – tingling right face	0.5 – tingling right hand	−1.5	99
	**1**	2.5 – tingling right face	0.8 – tingling right hand	−1.7	
	**2**	4.0 – pulling right face	1.5 – tingling right hand	−2.5	
	**3**	6.0 – pulling right face	0.8 – tingling right hand	−5.2	
	**Impedance**	848 Ω	Postop – 915 Ω; Chronic - 730 Ω		
	**Current density**	5.49 µC/cm^2^/phase	6.16 µC/cm^2^/phase		
**Patient 3**	**0**	2.6 – bilateral arm tingling	1 – tingling hand	−2.5	47
	**1**	4.1 – tingling right arm	1 – tingling hand	−4.0	
	**2**	6.0 – no side effects	0.3 – tingling hand	−5.7	
	**3**	6.0 – no side effects	3.3 – tingling hand	−2.7	
	**Impedance**	1099 Ω	Postop - 1343 Ω; Chronic - 658 Ω		
	**Current density**	10.55 µC/cm^2^/phase	7.13 µC/cm^2^/phase		

### Lead Location and Ventricular Size

Considering the standard error of deviation (set at 1.4 mm) when measuring lead location, long-term postoperative lead positions were slightly different across the cohort. The DBS leads were overall located at a more lateral position in recent scans in cases 2 and 3 (Δ = 2.68 and 1.88, respectively), as compared to the initial measurements. In case 2, the collar angle changed from 13 to 15, while in the two other cases, the arc and collar angles remained the same. When comparing atrophy indexes from the initial and most recent brain scans, there was a substantial change in the 3^rd^ ventricular width (120%), Evans index (6%), ventricular index (5%), and cella media index (17%) across the patients (as shown in Table 3).

**Table 3:** Two time-point comparison of percentage change in brain atrophy measurements and lead measurements.

	3^rd^ Ventricle Width	Evans index	Ventricular Index	Cella Media Index	Δ AP	Δ Lateral	Δ Axial
**Patient 1**	300%	5.7%	7.5%	5.5%	0.32	1.2	0.16
**Patient 2**	37.3%	9.4%	6.6%	15.1%	0.1	2.68	0.5
**Patient 3**	22.5%	4.1%	1.1%	29.1%	0.52	1.88	0.26
**Mean**	120%	6%	5%	17%	**-**	**-**	**-**

Change in lead position was measured using a distance formula: √ (X_pre_−X_post_)^2^+(Y_pre_−Y_post_)^2^+(Z_pre_−Z_post_)^2^. AP = antero-posterior.

## Discussion

We present a series of three cases with long-term DBS related management issues and an unexpected change in long-term programming thresholds as compared to the initial evaluation.

### Factors possibly underpinning the findings

Our patients presented with long-term issues after several years of DBS related stimulation-induced benefit. This presentation does not occur in typical surgery-related complications, since most of these issues are acute. Hardware-related complications should be considered as a potential factor for side effects or a loss of benefit, and these types of complications have been reported in 4.8% of cases [6], [14]. However, careful examination of the neurostimulator, impedances, as well associated current drains revealed no issues. In addition, plain x-rays of the DBS system were also normal, excluding a DBS lead/wire break. Another important issue could have been impedance variability [15]. However, the impedances across all cases decreased over time, and the values remained within reasonably normal ranges (Table 2), and these would therefore be unlikely to have resulted in the large changes in programming thresholds.

Lead migration was another possible hardware-related factor. Small shifts in position could have contributed to induction of side effects or to the loss of benefit [16]. Perioperative brain shift could also account for differences in lead location [17]. It is important to consider that by comparing the lead location measurements, a lateral shift was documented in two of our patients. This lateral shift could at least in part be explained by atrophy [18].

### Brain and nuclei atrophy hypothesis

Stimulation-induced side effects result from the spread of electrical current into brain regions other than the region intended for stimulation. The most interesting finding in our patients was the unexpected reduced programming thresholds that were uncovered following long-term follow-up. DBS-related adverse effects at relatively low levels of stimulation could suggest a suboptimally placed DBS lead. Besides the slight lateral lead shift found in two of our cases, a substantial change in atrophy indices was also manifested. It is unknown how atrophy may affect lead location, but presumably there would be a relationship. Change in atrophy indices could theoretically affect lead position by simply slightly repositioning the DBS lead closer to surrounding structures.

**Figure 2:**
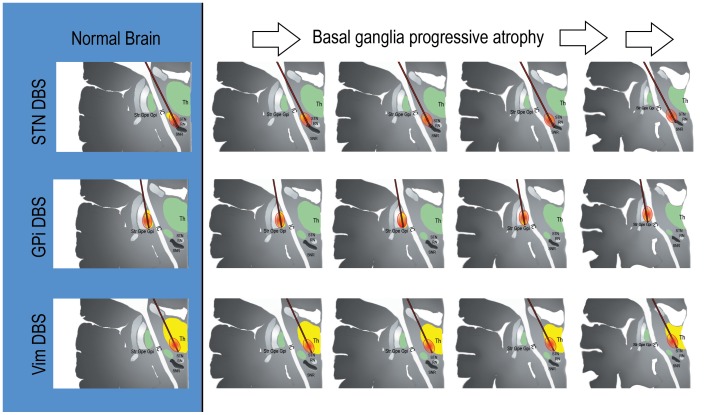
Theoretical figure including atrophy which may play a role in delayed stimulation induced side effects. Progressive 10%, 20%, 30%, and 40% atrophy with stimulation spread to other structures can be observed following columns to the right. DBS = Deep Brain Stimulation; Str = striatum; GPe = globus pallidus externus; GPi = globus pallidus internus; IC = internal capsule; Th = thalamus; STN = subthalamic nucleus; Vim = ventralis intermedius nucleus; RN = red nucleus; SNR = substantia nigra pars reticulata. This figure is theoretical and shows how atrophy may potentially affect lead location and stimulation fields.

Brain atrophy is expected with aging and is known to be hastened by neurodegeneration [19]. Annual rates of brain atrophy have been reported to be 0.32%/year in healthy adults [20] with a median volume loss of 10.35 ml/year in PD patients [21]–[23]. There has not been a standardized rate of brain atrophy for DBS patients reported. Regarding deep brain structures, thalamic and Vim total volumes are approximately 13000 mm^3^ and 218 mm^3^, respectively [24], and these decline over time have been reported in normal subjects [25], [26], and likely will also decline in affected subjects. Similarly, STN shrinks in volume with time [27]. We observed patients with an increase (worsening) in atrophy measurements (Table 2). The Vim's long narrow shape, measuring ∼8 mm (dorsal–ventral) by ∼3 mm (anterior–posterior) by ∼12 mm (medial–lateral) [28] and the typical trajectory of the DBS lead could be hypothesized to result in spreading current into an unintended and adjacent region (capsule or sensory areas). This effect could similarly occur in STN, considering its small size (150–240 mm^3^) [21], [29], and considering that STN atrophy and shifts in the lateral direction with increasing age [18], could also potentially result in spread of stimulation outside of the intended region. Atrophy could theoretically contribute to the clinically manifested symptoms and in the reduced programming threshold tolerances and subsequent decrement in current densities in two of our patients. The globus pallidus internus (GPi) is a larger structure (450 mm^3^), and its shape could possibly be more forgiving, but not immune to disease related atrophy and spread of stimulation [30]. No GPi DBS cases were observed in our clinic which actively follows over 600 patients, however a larger sample size and more careful follow-up will be needed. Figure 2 illustrates the DBS atrophy hypothesis, which would posit that similar amounts of electricity are delivered to a shrinking brain (assuming stability of impedance measurements). The mechanisms resulting in atrophy in this small group of patients is unknown and will need to be clarified in future studies which should include long-term cognition and other relevant measures. Future studies will need to measure nuclei atrophy with more sophisticated imaging, such as volumetry or tractography, and to compare to contralateral non-stimulated nuclei in unilateral cases.

### Other potential long-term issues

Disease progression could possibly explain the worsening of symptoms over time, as was observed in cases 2 and 3. However, assessment of the UPDRS and TRS motor scores suggested that the symptoms remained well controlled with chronic stimulation over time. Although worsening of tremor in ET patients with long-term follow-up is expected, and has been previously observed to be related to disease progression [31], the appearance of sensory side effects at lower voltages would suggests a different cause. Motor side effects, as in case 1, could have been a manifestation of dystonia, rather than internal capsule current spread. This notion though possible, was unlikely, since the symptoms continued to worsen with increasing stimulation voltage, and the symptoms abated when turning off the DBS. Additionally, there were programming threshold changes across all four DBS lead contacts.

In conclusion, unexpected emergent stimulation induced side effects or loss of benefit in DBS cases can be potentially important clinical issues that may manifest more frequently in longer-term DBS management. Brain atrophy and disease progression may possibly play a role in at least some of these patients; however, changes in lead position, and long-term pathophysiological changes cannot be completely ruled out. Clinicians should be aware that in DBS therapy there is delivery of electricity to a changing brain and that several factors may affect the response to long-term stimulation. It is therefore very important for DBS patients to have close follow-up and monitoring even after many years post-implant.
